# Growth Differentiation Factor 15 Predicts Cardiovascular Events in Peripheral Artery Disease

**DOI:** 10.3390/biom15070991

**Published:** 2025-07-11

**Authors:** Ben Li, Farah Shaikh, Houssam Younes, Batool Abuhalimeh, Abdelrahman Zamzam, Rawand Abdin, Mohammad Qadura

**Affiliations:** 1 Division of Vascular Surgery, St. Michael’s Hospital, Unity Health Toronto, University of Toronto, Toronto, ON M5B 1W8, Canada; benx.li@mail.utoronto.ca (B.L.);; 2 Department of Surgery, University of Toronto, Toronto, ON M5S 1A1, Canada; 3 Temerty Centre for Artificial Intelligence Research and Education in Medicine (T-CAIREM), University of Toronto, Toronto, ON M5S 1A1, Canada; 4 Institute of Medical Science, University of Toronto, Toronto, ON M5S 1A1, Canada; 5 Heart, Vascular, & Thoracic Institute, Cleveland Clinic Abu Dhabi, Abu Dhabi 112412, United Arab Emirates; 6 Department of Medicine, McMaster University, Hamilton, ON L8S 4L8, Canada; 7 Li Ka Shing Knowledge Institute, St. Michael’s Hospital, Unity Health Toronto, University of Toronto, Toronto, ON M5B 1W8, Canada

**Keywords:** growth differentiation factor 15, major adverse cardiovascular events, prognosis, peripheral artery disease

## Abstract

Peripheral artery disease (PAD) is associated with an elevated risk of major adverse cardiovascular events (MACE). Despite this, few reliable biomarkers exist to identify patients at heightened risk of MACE. Growth differentiation factor 15 (GDF15), a stress-responsive cytokine implicated in inflammation, atherosclerosis, and thrombosis, has been broadly studied in cardiovascular disease but remains underexplored in PAD. This study aimed to evaluate the prognostic utility of GDF15 for predicting 2-year MACE in PAD patients using explainable statistical and machine learning approaches. We conducted a prospective analysis of 1192 individuals (454 with PAD and 738 without PAD). At study entry, patient plasma GDF15 concentrations were measured using a validated multiplex immunoassay. The cohort was followed for two years to monitor the occurrence of MACE, defined as stroke, myocardial infarction, or death. Baseline GDF15 levels were compared between PAD and non-PAD participants using the Mann–Whitney U test. A machine learning model based on extreme gradient boosting (XGBoost) was trained to predict 2-year MACE using 10-fold cross-validation, incorporating GDF15 and clinical variables including age, sex, comorbidities (hypertension, diabetes, dyslipidemia, congestive heart failure, coronary artery disease, and previous stroke or transient ischemic attack), smoking history, and cardioprotective medication use. The model’s primary evaluation metric was the F1 score, a validated measurement of the harmonic mean of the precision and recall values of the prediction model. Secondary model performance metrics included precision, recall, positive likelihood ratio (LR+), and negative likelihood ratio (LR-). A prediction probability histogram and Shapley additive explanations (SHAP) analysis were used to assess model discrimination and interpretability. The mean participant age was 70 ± SD 11 years, with 32% (*n* = 386) female representation. Median plasma GDF15 levels were significantly higher in PAD patients compared to the levels in non-PAD patients (1.29 [IQR 0.77–2.22] vs. 0.99 [IQR 0.61–1.63] pg/mL; *p* < 0.001). During the 2-year follow-up period, 219 individuals (18.4%) experienced MACE. The XGBoost model demonstrated strong predictive performance for 2-year MACE (F1 score = 0.83; precision = 82.0%; recall = 83.7%; LR+ = 1.88; LR− = 0.83). The prediction histogram revealed distinct stratification between those who did vs. did not experience 2-year MACE. SHAP analysis identified GDF15 as the most influential predictive feature, surpassing traditional clinical predictors such as age, cardiovascular history, and smoking status. This study highlights GDF15 as a strong prognostic biomarker for 2-year MACE in patients with PAD. When combined with clinical variables in an interpretable machine learning model, GDF15 supports the early identification of patients at high risk for systemic cardiovascular events, facilitating personalized treatment strategies including multidisciplinary specialist referrals and aggressive cardiovascular risk reduction therapy. This biomarker-guided approach offers a promising pathway for improving cardiovascular outcomes in the PAD population through precision risk stratification.

## 1. Introduction

Peripheral artery disease (PAD), characterized by atherosclerotic narrowing of the lower limb arteries, impacts over 200 million individuals globally [1]. As described by Chioncel et al. (2019), the diagnosis of PAD is based on clinical data, functional tests, and imaging methods, while management options vary depending on the location and severity of the lesions and the chronic or acute nature of the disease [2]. While PAD is often associated with limb complications, including amputation, most affected individuals die from major adverse cardiovascular events (MACE), including myocardial infarction (MI) and stroke [3]. This elevated risk is largely due to the frequent co-occurrence of PAD with other atherosclerotic conditions, notably coronary artery disease (CAD) and cerebrovascular disease (CVD) [4]. Common risk factors, including advanced age, hypertension, diabetes mellitus, dyslipidemia, and smoking, contribute to this widespread vascular pathology observed in PAD patients [5]. Because PAD reflects a systemic vascular disease process, identifying those at highest risk for adverse systemic cardiovascular outcomes is essential [5]. Effective risk stratification enables earlier, coordinated intervention and the adoption of intensive medical management strategies to reduce cardiovascular risk [6]. One emerging strategy to refine prognostic assessment involves the identification of novel blood-based biomarkers [7,8,9]. Although our team has previously identified circulating biomarkers linked to limb outcomes in PAD [10], research aimed at identifying biomarkers that can predict broader cardiovascular events within this population remains sparse and warrants further investigation.

Growth differentiation factor 15 (GDF15), part of the transforming growth factor-beta (TGF-β) superfamily, is a stress-responsive cytokine implicated in various physiological and pathological processes [11]. It plays roles in modulating inflammation, maintaining metabolic balance, and contributing to endothelial dysfunction and atherosclerosis [11]. Over the past decade, GDF15 has emerged as a promising biomarker for cardiovascular disease [12,13]. Elevated blood concentrations of GDF15 have been associated with increased risk of CAD, adverse cardiac remodeling, and progression to heart failure [14,15]. Additionally, Negishi et al. (2021) showed that GDF15 predicts stroke events and death [16]. Despite extensive evaluation of GDF15 in CAD and CVD, its prognostic relevance in PAD has not been thoroughly investigated [14,15,16]. Considering the shared underlying mechanisms across PAD, CAD, and CVD, including endothelial dysfunction, dysregulated metabolism, and systemic atherosclerosis [4], examining the role of GDF15 in predicting systemic cardiovascular risk, specifically in the PAD population, represents a critical and underdeveloped area of research.

Previous studies investigating the prognostic significance of protein biomarkers in PAD have often been limited by their reliance on traditional statistical techniques and the omission of key clinical variables [17,18,19,20]. For example, De Haan et al. (2017) showed that GDF15 was associated with major amputation and mortality in patients with PAD [17]. However, they did not focus on incorporating GDF15 with relevant clinical features to build a prognostic model, and they did not assess the association between GDF15 and systemic cardiovascular events in patients with PAD [17]. Given the biological complexity of PAD, which is shaped by intricate interactions among metabolic pathways, clinical factors, and circulating proteins, we hypothesized that integrating biomarker profiles with patient-level clinical data could lead to more robust and accurate risk predictions than the use of protein markers alone [21]. Furthermore, the use of explainable machine learning offers a promising avenue to develop predictive models that are not only accurate but also interpretable and clinically actionable for forecasting systemic cardiovascular events in individuals with PAD [22,23,24].

This study assessed the predictive value of circulating GDF15 levels for MACE in patients with PAD by employing both interpretable statistical and machine learning approaches. By integrating GDF15 with key clinical variables, we aimed to construct prognostic models that are both accurate and clinically meaningful. These models could play an important role in improving early cardiovascular risk assessment among PAD patients, allowing for the timely identification of individuals at heightened MACE risk. Ultimately, such stratification may facilitate the use of more intensive medical/surgical therapies to mitigate the risk of MACE and improve overall cardiovascular outcomes in this vulnerable population.

## 2. Materials and Methods

### 2.1. Ethics

Ethical approval for this study was obtained from the Unity Health Toronto Research Ethics Board on 8 February 2017 (REB #16-375). Prior to enrollment, written informed consent was provided by all participants. All study activities were conducted in full accordance with the ethical principles set forth in the Declaration of Helsinki [25].

### 2.2. Design

This study was designed as a prognostic investigation, and its reporting adheres to the TRIPOD+AI guidelines for transparent development and validation of multivariable prediction models incorporating artificial intelligence [26].

### 2.3. Patient Recruitment

Between September 2020 and February 2022, patients with and without PAD were prospectively enrolled from ambulatory clinics at our institution. PAD was diagnosed based on an ankle–brachial index (ABI) below 0.9 or a toe–brachial index (TBI) below 0.67, in conjunction with absent or diminished pedal pulses [27]. Individuals classified as non-PAD exhibited an ABI of 0.9 or greater, a TBI of at least 0.67, and normal pedal pulses [27]. Patients were excluded if they presented with acute limb ischemia, had experienced an acute coronary syndrome, or showed elevated troponin levels within the previous three months.

### 2.4. Baseline Characteristics

Baseline demographic and clinical information was collected for all participants, including age, sex, smoking history (both current and former), and comorbid conditions including hypertension, diabetes, dyslipidemia, congestive heart failure (CHF), coronary artery disease (CAD), and prior stroke or transient ischemic attack (TIA). Hypertension was defined by a systolic blood pressure of 130 mmHg or higher, a diastolic blood pressure of at least 80 mmHg, or ongoing use of antihypertensive medications. Dyslipidemia was identified by triglyceride concentrations greater than 1.7 mmol/L, total cholesterol levels exceeding 5.2 mmol/L, or current lipid-lowering therapy. Diabetes mellitus was determined by an HbA1c threshold of 6.5% or above or active treatment with antidiabetic agents. Additionally, data on cardiovascular preventive medications, including acetylsalicylic acid (ASA), statins, angiotensin-converting enzyme inhibitors (ACE-I), and angiotensin receptor blockers (ARB), were recorded. Definitions of cardiovascular risk factors followed the guidelines set forth by the American College of Cardiology [28,29].

### 2.5. Plasma GDF15 Concentration Measurement

At recruitment, blood samples were obtained from study participants, and plasma GDF15 levels were quantified in duplicate using a commercially available LUMINEX assay kit (Bio-Techne, Minneapolis, MN, USA) [30]. Before sample analysis, the MagPix system (Luminex Corp., Austin, TX, USA) [31] was calibrated with Fluidics Verification and Calibration bead kits (Luminex Corp., Austin, TX, USA) [32]. To reduce assay variability, all measurements were conducted on the same day. The intra-assay and inter-assay coefficients of variation were maintained below 10%. A minimum of 50 beads per sample were captured and analyzed using Luminex xPonent software version 4.3 [33].

### 2.6. Follow-Up and Outcomes

Participants attended routine follow-up visits at one and two years after their baseline assessment, with additional appointments arranged as clinically necessary. The primary outcome was the occurrence of MACE within the 2-year follow-up period. MACE was defined as a composite endpoint including MI, stroke, or mortality, determined through direct patient follow-up. MI diagnosis required evidence of a rise and/or fall in cardiac troponin levels, with at least one measurement above the 99th percentile upper reference limit, plus one or more of the following: (a) new ischemic changes on an electrocardiogram, (b) symptoms indicative of myocardial ischemia, (c) the presence of pathological Q waves, (d) imaging revealing new loss of viable myocardium or regional wall motion abnormalities consistent with ischemia, or (e) confirmation of a coronary thrombus by angiography or autopsy [34]. This definition follows the guidelines from the American Heart Association, the American College of Cardiology, the European Society of Cardiology, and the World Heart Federation [34]. Stroke was characterized as the death of brain, spinal cord, or retinal tissue caused by ischemia, identified either by (a) pathological, imaging, or other objective evidence of localized ischemic injury in a vascular territory, or (b) clinical symptoms of focal ischemia lasting 24 h or longer or resulting in death, with exclusion of alternative diagnoses [35]. These criteria align with the standards from the American Stroke Association and the American Heart Association [35]. Death was classified as all-cause mortality.

### 2.7. Model Development and Evaluation

Extreme gradient boosting (XGBoost) was selected as the predictive modeling approach. XGBoost is an ensemble learning technique that builds a strong classifier by combining multiple decision trees [36]. Decision trees operate by partitioning data into branches based on feature values, allowing the model to predict outcomes by examining various covariates [37]. As a non-parametric method, XGBoost effectively manages large, high-dimensional datasets [36]. It is also known for its computational efficiency and scalability, achieved through parallel processing, and incorporates regularization techniques to reduce overfitting and enhance model generalizability [36]. This algorithm was chosen due to its widespread application in the literature for classification and regression tasks and its proven ability to deliver high predictive accuracy in clinical outcome modeling using structured data [38,39,40].

The dataset was randomly split into training and testing subsets, with 70% of the data used for model training and the remaining 30% reserved for evaluation. The XGBoost model was trained to predict 2-year MACE using 10-fold cross-validation. Input variables comprised clinical factors including age, sex, hypertension, dyslipidemia, diabetes, smoking status (current or former), CHF, CAD, prior stroke/TIA, and medication use (ASA, statins, and ACE-I/ARB), as well as plasma GDF15 concentrations. It is important to note that the analysis included selected clinical features, but not all possible risk factors that could potentially influence the predictive accuracy of the model. After training, model performance was assessed on the independent test set.

### 2.8. Statistical Analysis

Baseline characteristics were summarized using descriptive statistics. Continuous variables were expressed as the mean ± standard deviation and compared between groups using the Mann–Whitney U test due to non-normal data distributions. Categorical variables were presented as counts and percentages and analyzed using Chi-square tests. Circulating GDF15 levels were also compared between groups using the Mann–Whitney U test. Two-year event rates were compared between PAD and non-PAD cohorts via Chi-square analysis. The primary metric for evaluating the predictive model was the F1 score, which represents the harmonic mean of precision and recall for 2-year MACE prediction [41]. The F1 score is computed as 2 × [(precision × recall) ÷ (precision + recall)], ranging from 0 (no precision or recall) to 1 (perfect precision and recall) [41]. Secondary performance measures included precision, recall, positive likelihood ratio (LR+), and negative likelihood ratio (LR−). The optimal classification threshold was determined by maximizing Youden’s Index derived from receiver operating characteristic curve analysis [42]. Prediction histograms were generated to visually assess the model’s discrimination ability between patients who did vs. did not experience 2-year MACE. To enhance interpretability, SHAP (Shapley additive explanations) analysis quantified the contribution of each feature to the model predictions [43]. Statistical significance was set at a two-sided *p*-value of less than 0.05. All analyses and model training/evaluations were performed using Python version 3.13.3 [44].

## 3. Results

### 3.1. Patients

The study included a total of 1192 participants, consisting of 454 individuals diagnosed with PAD, all presenting with intermittent claudication, and 738 without PAD. Patients with PAD were notably older, with an average age of 71.11 ± 9.64 years, compared to 68.92 ± 11.29 years in the non-PAD group (*p* = 0.003). The sex distribution was comparable between groups, with males representing 69.4% of the PAD cohort and 66.5% of the non-PAD cohort, a difference that was not statistically significant (*p* = 0.338). Comorbid conditions were more frequently observed among those with PAD. Specifically, hypertension affected 83.3% of PAD patients versus 64.8% in the non-PAD group (*p* < 0.001), while dyslipidemia was reported in 82.4% of the PAD group compared to 64.5% of controls (*p* < 0.001). Diabetes prevalence was also higher in the PAD cohort at 42.3%, relative to 25.6% in the non-PAD group (*p* < 0.001). Regarding tobacco use, current smoking was more common in PAD patients (34.3%) than in those without PAD (29.8%), reaching statistical significance (*p* = 0.001). Conversely, former smoking was more prevalent among non-PAD individuals (70.2%) compared to PAD patients (65.7%), which was also significant (*p* = 0.015). When examining cardiovascular history, CAD was present in 39.2% of PAD patients, surpassing the 29.3% prevalence in the non-PAD group (*p* = 0.005). Similarly, prior stroke or TIA was more frequently reported among PAD patients (18.1%) than non-PAD participants (13.1%; *p* = 0.026). No statistically meaningful difference was found in CHF rates between the groups (6.2% vs. 8.5%; *p* = 0.166). Patterns of medication usage aligned with the higher cardiovascular risk profile of PAD patients. Statins were prescribed more often in the PAD group (76.4%) compared to in non-PAD individuals (61.0%; *p* < 0.001). Use of ACE-I/ARB was similarly elevated among PAD patients (57.5% vs. 38.1%; *p* < 0.001). ASA use was also more common in those with PAD (76.9%) than in the non-PAD group (56.6%; *p* < 0.001) (Table 1).

### 3.2. Plasma GDF15 Levels

Median plasma GDF15 concentrations differed significantly between groups. In participants without PAD, the median level was 0.99 pg/mL (interquartile range [IQR] 0.61 to 1.63), whereas those with PAD exhibited a higher median concentration of 1.29 pg/mL (IQR 0.77 to 2.22). This difference in GDF15 levels between the two groups was statistically significant (*p* < 0.001).

### 3.3. Outcomes

Over the 2-year follow-up period, MACE occurred in 115 PAD patients (25.3%) and 104 non-PAD patients (14.1%), with a statistically significant difference (*p* < 0.001). MI occurred in 99 PAD patients (21.8%) and in 89 non-PAD patients (12.1%), with a significant difference (*p* < 0.001). Stroke was reported in 24 PAD patients (5.3%) compared to 17 non-PAD patients (2.3%) (*p* = 0.009). Death occurred in 15 PAD patients (3.3%) and in 16 non-PAD patients (2.2%), with no statistically significant difference (*p* = 0.312) (Table 2).

### 3.4. Model Performance for Predicting 2-Year MACE

**Figure 1:**
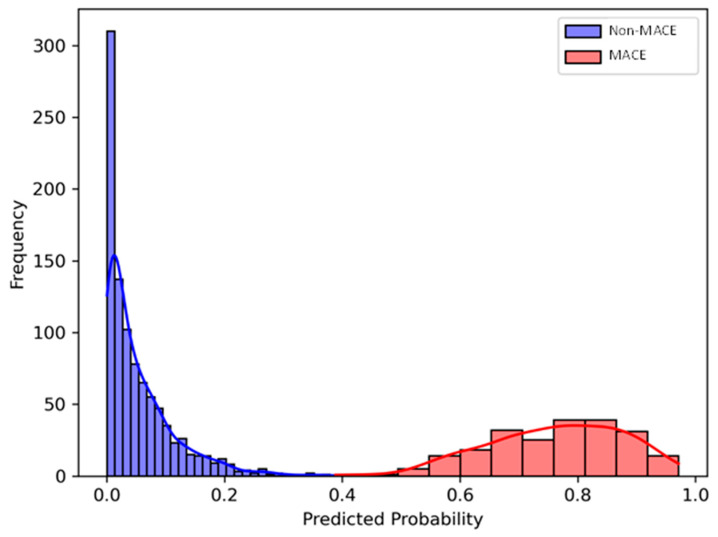
Predicted probability histogram for 2-year major adverse cardiovascular events (MACE) using the extreme gradient boosting (XGBoost) model incorporating demographic/clinical features and plasma growth differentiation factor 15 (GDF15) levels. There is a clear separation in predicted risk scores between patients with 2-year MACE (red) and patients without 2-year MACE (blue).

The performance of the XGBoost model for predicting 2-year MACE using circulating GDF15 levels and demographic/clinical features at the optimal cut-off probability of 0.196 based on Youden’s Index were as follows: F1-score of 0.83, precision of 82.0%, and recall of 83.7%. At this threshold, the model achieved an LR+ of 1.88 and an LR− of 0.83. The prediction probability histogram for 2-year MACE showed strong separation between patients who developed MACE and those who did not, based on prediction probabilities generated by the XGBoost model. Among the non-MACE group, the predicted probabilities were heavily concentrated near zero, with the highest frequency observed below 0.1. In contrast, patients who experienced MACE displayed predicted probabilities largely ranging from 0.6 to 0.85. This clear distinction in predicted probability distributions indicates that the model was highly effective at differentiating between patients who develop vs. do not develop 2-year MACE (Figure 1).

### 3.5. SHAP Analysis of XGBoost Model for Explainability

**Figure 2:**
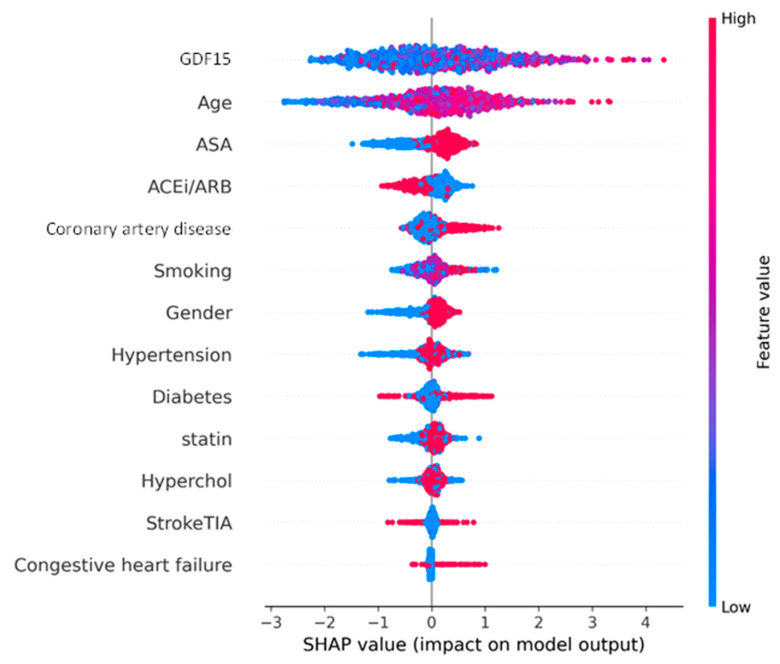
Shapley additive explanations (SHAP) beeswarm plot showing feature contributions to the prediction of 2-year major adverse cardiovascular events (MACE). Each point represents one patient; the position on the x-axis shows the impact on the model’s output, and the color indicates the feature value (red = high, blue = low). GDF15 (growth differentiation factor 15); ASA (acetylsalicylic acid); hyperchol (dyslipidemia); ACEi (angiotensin-converting enzyme inhibitor); ARB (angiotensin II receptor blocker); StrokeTIA (previous stroke or transient ischemic attack).

The SHAP beeswarm plot for MACE shown in Figure 2 provides detailed insight into the impact of each predictor variable on the model’s output. GDF15 was the strongest contributor to the prediction of 2-year MACE. High GDF15 values were consistently associated with a positive SHAP value, meaning that they increased the model’s probability for predicting the development of 2-year MACE. The SHAP values for GDF15 reached as high as +4, with a consistent rightward pull for higher GDF15 levels, indicating a very strong effect on 2-year MACE prediction in most patients. Age was the second most influential feature, with older patients also shifting predictions toward higher MACE risk. ASA and ACE-I/ARB use also contributed, with high values tending to increase the prediction probability of 2-year MACE, which may reflect their association with higher risk individuals who are more likely to be prescribed cardiovascular risk reduction medications. CAD and smoking contributed to a lesser but still relevant extent, while features such as dyslipidemia, diabetes, and hypertension showed smaller effects, with most SHAP values close to zero.

**Figure 3:**
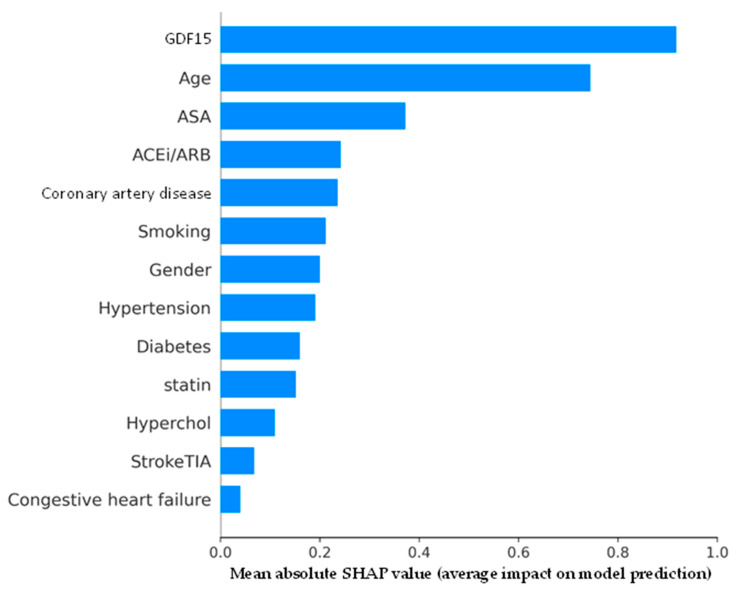
Shapley additive explanations (SHAP) bar plot displaying mean absolute SHAP values for each feature in the 2-year major adverse cardiovascular event (MACE) prediction model. GDF15 (growth differentiation factor 15); ASA (acetylsalicylic acid); hyperchol (dyslipidemia); ACEi (angiotensin-converting enzyme inhibitor); ARB (angiotensin II receptor blocker); StrokeTIA (previous stroke or transient ischemic attack).

The SHAP summary plot in Figure 3 quantified the average importance of each feature in the prediction model by calculating the mean absolute SHAP values. GDF15 had the highest average SHAP value at 0.917, indicating it was the most influential predictor for 2-year MACE. This was followed by age (0.744), contributing substantially to the model’s output. ASA (0.372), ACE-I/ARB (0.242), and CAD (0.236) were also key predictors. Smoking status (0.212) and gender (0.200) played meaningful roles, while hypertension (0.191), diabetes (0.159), and statin use (0.152) contributed to a lesser extent. Dyslipidemia (0.109), previous stroke/TIA (0.068), and CHF (0.040) exerted a relatively minor influence on the model’s predictions.

## 4. Discussion

### 4.1. Summary of Findings

In this study, we applied interpretable statistical and machine learning methods to identify GDF15 as a prognostic biomarker for systemic cardiovascular events in patients with PAD. By combining plasma GDF15 measurements with demographic and clinical variables, we developed a robust predictive model that accurately forecasts 2-year MACE in PAD patients. Several key observations emerged from our analysis. Firstly, individuals with PAD exhibited elevated plasma GDF15 levels compared to those without PAD. Secondly, our machine learning model, which integrated GDF15 data with clinical characteristics, achieved strong predictive performance for 2-year MACE, with an F1 score of 0.83. Thirdly, a probability distribution plot clearly differentiated patients who experienced 2-year MACE from those who did not, underscoring the model’s clinical relevance. Finally, explainability analysis using SHAP values identified GDF15 as the most influential predictor, surpassing traditional risk factors such as age, cardiovascular history, medication use, and smoking status. Together, these findings highlight the utility of GDF15 in enhancing cardiovascular risk stratification for PAD patients, potentially informing more precise medical/surgical treatment strategies to improve cardiovascular outcomes. Given these encouraging prognostic insights, further basic and translational studies are needed to elucidate GDF15’s role in cardiovascular disease pathophysiology and to evaluate its viability as a therapeutic target for reducing systemic cardiovascular risk in patients with PAD.

### 4.2. Comparison with Existing Literature

Previous research has investigated the association of GDF15 with PAD-related outcomes. For example, De Haan et al. (2017) found that elevated GDF15 levels correlated with major amputation and mortality in patients with PAD [17]. However, there are important distinctions between our studies. First, De Haan et al. focused specifically on major amputation and death, whereas our study evaluated the relationship between GDF15 and a broader composite cardiovascular outcome, MACE [17]. Since MACE represent the leading cause of mortality in PAD patients, our results complement and extend the limb-specific findings of De Haan et al. by emphasizing the systemic cardiovascular relevance of GDF15 in the PAD population [17]. Second, while their cohort predominantly included patients with chronic limb-threatening ischemia, our study primarily enrolled patients with intermittent claudication, a less advanced stage of PAD [17]. Patients with chronic limb-threatening ischemia often require urgent interventions, limiting the window for preventive risk management [17]. In contrast, our focus on patients with less severe disease aims to facilitate earlier risk stratification to improve cardiovascular outcomes. Third, De Haan et al. employed conventional statistical methods to focus on assessing GDF15 as a single biomarker, whereas we applied explainable machine learning techniques to integrate GDF15 with multiple clinically relevant features, resulting in a highly accurate predictive model for 2-year MACE [17]. Thus, while their work identified an association between GDF15 and limb-related outcomes, our study advances prognostication by combining GDF15 with relevant clinical features to predict broader systemic cardiovascular events with greater potential for clinical utility [17]. Elsewhere, Chuang et al. (2025) demonstrated that elevated GDF15 was associated with an increased risk of developing PAD among patients with diabetes [45]. Consistent with this, our findings showed that circulating GDF15 levels were elevated in both diabetic and non-diabetic PAD patients compared to the results for non-PAD patients. The prognostic value of GDF15 for systemic cardiovascular events in patients with atherosclerotic cardiovascular disease including CAD and CVD has also been previously demonstrated in an individual patient-level meta-analysis by Kato et al. (2023) [46]. Collectively, our findings align with the existing literature and expand it by incorporating explainable machine learning models that combine GDF15 measurements with clinical data to accurately predict systemic cardiovascular outcomes in PAD patients. This body of work underscores the importance of further mechanistic research to elucidate GDF15’s role in the intersection of PAD, CAD, and CVD. Ultimately, these insights could facilitate enhanced risk stratification and lead to the development of novel targeted therapies aimed at improving cardiovascular outcomes in the PAD population.

### 4.3. Explanation of Findings

The association between elevated GDF15 levels and increased MACE risk in PAD patients may be explained through several biological pathways. The gene for GDF15 is found on chromosome 19p13.11, located on the forward DNA strand and flanked by the pyroglutamyl-peptidase I and leucine-rich repeat-containing 25 (LRRC25) genes [47]. GDF15 is first produced as an inactive precursor protein, pro-GDF15, which forms dimers through cysteine bonds [48]. This pro-form undergoes cleavage at a conserved RXXR amino acid sequence, resulting in a mature 112-amino acid dimeric protein and a cleaved pro-peptide fragment [48]. The mature form possesses cysteine residues that create a fourth intrachain disulfide bond [48]. After processing, the dimeric protein, approximately 25 kilodaltons in size and stabilized by a single interchain disulfide bond, is released into the extracellular matrix and circulates in human plasma [48]. Clinically, elevated circulating GDF15 levels have been reported in individuals exhibiting vascular dysfunction, atherosclerosis, and thrombotic disorders [49]. At the cellular level, increased expression of GDF15 has been identified in subendothelial macrophages within atherosclerotic plaques, co-localizing with oxidized low density lipoprotein-laden macrophages and inflammatory mediators [50]. In the context of cardiac injury, GDF15 levels rise sharply in infarcted myocardium compared to the levels in healthy tissue, and plasma concentrations increase following acute myocardial infarction [51]. The biomarker’s levels also correlate with the severity of chronic heart failure [52]. Supporting these findings, experimental mouse models show a near 20-fold increase in myocardial GDF15 mRNA expression following MI compared to the levels in uninjured hearts [53]. In the CVD literature, Mihalovic et al. (2024) showed that elevated GDF15 is associated with stroke severity, myocardial injury, and poor clinical outcomes in patients after acute ischemic stroke [54]. Additionally, GDF15 has emerged as a predictor of chronic kidney disease progression and renal function deterioration in patients with preexisting renal pathology [55]. It also forecasts acute kidney injury after cardiac surgery [56]. The widespread involvement of GDF15 in diverse cardiovascular and systemic pathophysiological processes, including inflammation, metabolic dysregulation, atherosclerosis, and endothelial dysfunction, likely explains its strong prognostic association with systemic cardiovascular events in patients with PAD, which is consistent with our study findings [47]. Preliminary findings from our SHAP analysis demonstrated that higher levels of GDF15 are associated with poorer cardiovascular prognosis in patients with PAD; however, additional confirmatory studies are needed to further characterize this relationship.

### 4.4. Implications

These findings carry important implications for the clinical care of patients with PAD. By integrating GDF15 levels with clinical variables in a machine learning-based prediction model, our approach provides actionable insights to inform MACE risk management. Incorporating plasma GDF15 measurements into routine clinical practice could enhance the ability of healthcare providers to stratify PAD patients based on their MACE risk. This strategy is particularly valuable in primary care settings, where early recognition of high-risk patients enables proactive, tailored management [57]. General practitioners could implement plasma GDF15 measurement as part of routine bloodwork for individuals with PAD [57]. Patients flagged as high risk could be referred promptly to a multidisciplinary team, including cardiologists, vascular surgeons, and neurologists, for more in-depth assessment and individualized care planning [58]. In contrast, those deemed as low risk may continue to be managed by their primary care provider, with a focus on guideline-directed therapies such as antiplatelets, statins, and lifestyle modification [59]. For those referred to specialists, personalized and risk-adapted strategies can be applied. For instance, combining low-dose rivaroxaban with ASA has been shown to reduce cardiovascular events in patients with stable PAD or CAD [60]. High-risk individuals may also benefit from advanced imaging, such as coronary or cerebral angiography, to diagnose and treat subclinical but hemodynamically significant lesions before complications arise [61]. In summary, the clinical integration of GDF15 offers a pathway toward more individualized prognostication and management of systemic cardiovascular risk in PAD. This precision-driven model of care could improve cardiovascular outcomes, optimize specialist referrals, and support more effective healthcare resource utilization [62]. Despite the high predictive effectiveness of the model, additional investigation is needed to determine how these results should be used in everyday clinical practice, including whether GDF-15 should be routinely measured and what interventions would be recommended for patients with elevated levels of this biomarker.

### 4.5. Limitations

This study has several limitations that should be acknowledged when interpreting the results. First, the research was conducted within a single academic institution, which may limit the applicability of the results to other populations, especially those with different demographic or clinical characteristics. Broader validation in multicenter settings is necessary to confirm generalizability to more diverse populations. Second, the analysis was confined to two years of follow-up, which may not fully capture the long-term prognostic utility of GDF15 in chronic vascular conditions such as PAD, CAD, and CVD. Extended follow-up is needed to assess the durability of GDF15’s predictive performance over time. Third, the study did not have sufficient statistical power to assess the relationship between GDF15 and the individual components of MACE. Future studies with larger patient cohorts and a greater number of events will be important to evaluate whether GDF15 can accurately predict specific cardiovascular outcomes including MI and stroke individually. Fourth, patients experiencing acute coronary syndrome, elevated troponin, or acute limb ischemia within the last 3 months were excluded. The focus was mainly on individuals with less advanced PAD (intermittent claudication), which may not reflect the full spectrum of patients with PAD. Further validation studies incorporating more diverse PAD cohorts are needed to confirm our findings. Finally, while GDF15 shows potential as a clinical biomarker, it is currently used primarily in research settings. Additional translational work is needed to assess the feasibility and cost-effectiveness of integrating plasma GDF15 measurements into routine clinical workflows for PAD management.

## 5. Conclusions

In this study, we leveraged interpretable statistical and machine learning methods to demonstrate the prognostic value of GDF15 for predicting 2-year MACE in patients with PAD. By combining plasma GDF15 concentrations with relevant clinical variables, we constructed a robust prognostic machine learning model capable of accurately identifying individuals with PAD at elevated systemic cardiovascular risk. This approach offers a meaningful advance in risk stratification for PAD patients and supports the implementation of personalized care strategies. Specifically, high-risk individuals identified through our algorithm may benefit from earlier and more intensive treatment strategies, including multidisciplinary cardiovascular specialist referrals and aggressive medical/surgical management to reduce the incidence of MI and stroke, the primary drivers of mortality in this population. Furthermore, these findings underscore the need for continued basic and translational investigations into the biological mechanisms linking GDF15 to cardiovascular disease pathophysiology. A deeper understanding of GDF15’s role in atherosclerosis and vascular dysfunction could ultimately inform the development of targeted treatments, enhancing our capacity to understand and address the overlapping pathophysiology of PAD, CAD, and CVD to improve cardiovascular outcomes.

## Figures and Tables

**Table 1:** Baseline characteristics of patients with and without peripheral artery disease.

	Non-PAD (*n* = 738)	PAD (*n* = 454)	*p*-Value
Age, years	68.92 ± 11.29	71.11 ± 9.64	0.003
Female sex	247 (33.5)	139 (30.6)	0.338
Hypertension	478 (64.8)	378 (83.3)	<0.001
Dyslipidemia	476 (64.5)	374 (82.4)	<0.001
Diabetes	189 (25.6)	192 (42.3)	<0.001
Smoking, past	337 (70.2)	241 (65.7)	0.015
Smoking, current	143 (29.8)	126 (34.3)	0.001
Congestive heart failure	63 (8.5)	28 (6.2)	0.166
Coronary artery disease	216 (29.3)	178 (39.2)	0.005
Previous stroke or transient ischemic attack	97 (13.1)	82 (18.1)	0.026
Statin	450 (61.0)	347 (76.4)	<0.001
ACE-I/ARB	281 (38.1)	261 (57.5)	<0.001
ASA	418 (56.6)	349 (76.9)	<0.001

Values are presented as mean ± standard deviation for continuous variables and frequency (percentage) for categorical variables. *p*-values are derived from Mann–Whitney U tests for continuous variables and Chi-square tests for categorical variables. Abbreviations used: SD—standard deviation; PAD—peripheral artery disease; ACE-I—angiotensin-converting enzyme inhibitor; ARB—angiotensin II receptor blocker; ASA—acetylsalicylic acid.

**Table 2:** Two-year major adverse cardiovascular events.

	Non-PAD (*n* = 738)	PAD (*n* = 454)	*p*-Value
Major adverse cardiovascular event	104 (14.1)	115 (25.3)	<0.001
Myocardial infarction	89 (12.1)	99 (21.8)	<0.001
Stroke	17 (2.3)	24 (5.3)	0.009
Death	16 (2.2)	15 (3.3)	0.312

Values are presented as frequency (percentage). *p*-values reflect between-group comparisons using Chi-square tests. Abbreviation: PAD—peripheral artery disease.

## Data Availability

The original contributions presented in the study are included in the article; further inquiries can be directed to the corresponding author.
